# Continued Medical Waste Exposure of Recyclable Collectors Despite Dumpsite Closures in Brazil

**DOI:** 10.5696/2156-9614-9.23.190905

**Published:** 2019-07-23

**Authors:** Tara Rava Zolnikov, Daisy Ramirez-Ortiz, Hayssa Moraes, Vanessa Resende Nogueira Cruvinel, Aldira Dominguez, Dayani Galato

**Affiliations:** 1 National University, San Diego, California, USA; 2 Florida International University, Miami, Florida, USA; 3 University of Brasilia, Brasilia, Brazil

**Keywords:** recyclable material collectors, recyclable collectors, healthcare waste, occupational health and safety, qualitative research, Brazil

## Abstract

**Background.:**

Brasilia, the capital of Brazil, currently has the largest dumpsite of the Americas at Estrutural, with over 30 million tons of waste accumulated. Recyclable waste collectors are a group of workers who, in addition to having a low socioeconomic status and residing in vulnerable areas, work sorting garbage in inadequate and unsanitary areas. This profession puts individuals at risk, resulting in death, mutilation, and disease for workers.

**Objectives.:**

The aim of this study was to understand the effects of waste on recyclable collectors, along with their perceptions of associated risks.

**Methods.:**

A qualitative study was conducted, using interviews with 34 participants at Estrutural.

**Results.:**

Collectors were exposed to several hazards, including biological, physical, and more extreme hazards (e.g. being run over by waste trucks). Personal protective equipment was not adequately used, exposing recyclable collectors to injury. Accidents included cuts, burns, skin lesions, eyes lesions, and arm, leg, head, feet, and hand injuries and amputations. Often, homecare remedies and collected medical waste (e.g. pain killers) were used on these injuries instead of seeking out proper medical care.

**Conclusions.:**

Recyclable collectors were aware of occupational hazards, but lacked education on the risks and consequences associated with exposure to medical hazards. Moreover, Brazil recently formally closed all dumpsites, complicating this issue. The findings of the present study confirm the need to address these hazards to provide a safe working environment for waste pickers.

**Participant Consent.:**

Obtained

**Ethics Approval.:**

This study was approved by the Research and Ethics Committee of the Health School of Brasília University under Opinion n. 1.517.670/2016.

**Competing Interests.:**

The authors declare no competing financial interests.

## Introduction

There are approximately 15 million people engaged in waste collection worldwide.^1^ In low- and middle-income countries, recyclable collectors represent 1% of the urban workforce.^1^,^2^ Data from two Latin American countries suggest that recyclable collectors account for 0.6% of the urban informal employment in Lima, Peru and 0.5% in Brazil.^2^ Although not recognized as a profession or as part of the waste management system in many countries, informal waste collection serves as a sustainable income generating activity that offers a livelihood for waste pickers and their families.^3^–^5^

Informal recyclable waste collection accounts for a significant percentage of urban employment in Brazil. The situation in Brazil provides insight into issues of waste management and recyclable waste collection for a number of reasons. First, Brazil is the only country that systematically collects statistical data on recyclable collectors because it formally legalized informal waste collection as a profession.^6^ ‘Recyclable material collector’ is a profession recognized by the Brazilian Ministry of Labor and Employment, which is tasked with guaranteeing the right to a safe environment for working individuals.^7^,^8^ Second, in 2010, the government created a strategy to dismantle dump sites.^5^,^9^ The aim was to decrease negative outcomes related to unsafe waste gathering techniques. However, even though many sites are now officially closed, they continue to be worked in an informal fashion, contributing to continued adverse health effects for workers.^5^ The final reason Brazil's waste management is important is because Brasilia, the capital of Brazil, currently has the largest dumpsite in the Americas, Estrutural. According to the Brazilian Association of Public Sanitation and Special Residue Companies, approximately 30 million tons of garbage have accumulated in the dumpsite of Estrutural. Since the mid 1960's, this site has received all solid waste produced in all the Federal District; per data provided by the technical directory of the Sanitation Department in 2014, this amount translates to about 860 000 tons of waste annually.^10^ Thus, despite the national policy on solid waste, the governance enforcing dumpsites closures in Brazil, many of these dumpsites continue to operate informally, including Estrutural, which was ‘closed’ at the end of 2017.^10^

The Brazilian national policy on solid waste has drawn more visibility to the recycling sector, allowing the creation of cooperatives and associations, and several programs for the recovery of recyclable waste.^11^ This situation can promote awareness and decrease adverse effects in affected populations; however, the working and living conditions of informal waste collectors have not actually improved, with the great majority operating without technical guidance from the local government and basic benefits of employment, and being poorly compensated for recovered materials.^12^ In fact, only 5% of waste collectors in Brazil have contracts and income above the national minimum average.^12^ In the Federal District dumpsite, there are about 1500 recyclable collectors who are part of six associations for recyclable materials, although it is estimated that 230 000 people work as recyclable collectors (of all types of waste) in Brazil.^13^ Although some progress has occurred, there is still a need to improve the health and working conditions of recyclable collectors in Brazil by strengthening health and safety standards.^14^

**Abbreviations**
*PPE*: Personal protective equipment

Recyclable collectors in the informal sector experience increased occupational health risks due to direct contact with waste, manual handling, and lack of personal protective equipment (PPE).^4^,^5^ Waste collection is linked to adverse health effects, including injuries (e.g. cuts), hearing loss, musculoskeletal disorders, respiratory diseases (e.g. bronchitis, pneumonia, allergies), skin diseases (e.g. dermatitis, sun burn), communicable diseases (e.g. HIV/AIDS, sexually transmitted infections, hepatitis B and C), waterborne diseases (e.g. dengue, leptospirosis, diarrhea), and psychological disorders (e.g. depression, stress, anxiety).^5^,^14^,^15^

One of the key contributors to this increased occupational risk is exposure to medical waste.^16^–^19^ Contact with medical waste improperly disposed of by the healthcare industry can result in exposure to biological, chemical, radioactive and sharp objects.^20^–^22^ Among the most common objects responsible for these accidents are syringes, needles, glasses, spikes, and objects that cause cuts and injuries. In Brazil, despite the mandatory implementation of the medical waste management plan in health-care settings, very few establishments currently comply with internal and external regulations for medical waste management.^23^ This lack of universal compliance with the medical waste management plan has resulted in 12.5% of medical waste being disposed in dump sites and mixed together with other types of waste.^23^ Consequently, this may be a cause of occupational accidents and diseases among informal waste collectors, who are at a higher risk (compared to the general population) of encountering inadequately disposed medical waste.^24^ Recyclable collectors are at risk of exposure either directly (e.g. syringe puncturing skin) or indirectly (e.g. environmental, occupational, and food factors).^25^

Previous research studies conducted in the recycling sector in Brazil have not typically focused on the perceptions of the risks and hazards of waste pickers regarding work in unregulated dumpsites and landfills. Since Brazil has recently officially closed all open-air dump sites, research could be used to better understand the health and safety risks associated with waste collection in these newly informal sites. This information may be essential to tailor the national policy on solid waste to incorporate the management of medical waste and integrate all groups of informal recyclable collectors, as well as to ensure adequate occupational health and safety standards in this work setting and improve the efficiency of waste collection methods in Brazil. Thus, the objective of this qualitative study was to understand recyclable collectors' risks, opinions, and perceptions regarding occupational hazards, with a focus on medical waste in the Federal District in Brazil.

## Methods

This study took place in 2016 in Brasilia, Brazil at the Federal District waste site of Aterro Controlado do Joquei. Participants were selected through convenience sampling, which was used to provide an adequate sample of person-time hours spent in the Federal District, age, and gender differences. Inclusion criteria were that individuals must be over 18 years of age and be present at the waste site during interviews. The present research was approved by the Research and Ethics Committee of the Health School of Brasília University under Opinion n. 1.517.670/2016; all participants gave full informed consent.

In this area, recyclable collectors have historically gathered recyclable materials to sell to recycling companies for profit. As such, the recyclable collectors are exposed to a variety of garbage, as they sift through waste in search of recyclable goods. Semi-structured interviews of recyclable collectors were used to understand the types of hazards associated with this recyclable waste collection.

Qualitative methods were used in this study. Qualitative methods are an important tool for researchers who are seeking to understand deeper cultural implications including multiple participant perspectives in varying contexts.^26^ A phenomenological study was specifically used, which seeks to understand the experiences of participants. The expectation was that by interviewing participants and teasing apart complexities amongst self, others, culture, and environment, a greater understanding of the perspectives of waste pickers regarding occupational health hazards would be uncovered.

In general, qualitative research must provide measures to ensure validity of the research. In this case, the researchers established trustworthiness through credibility, multiple participant perspectives, peer debriefing and review, reflexive journaling, and field notes. The interviews were conducted on an individual basis, which sought to provide a greater bond and trust between the researcher and the interviewee, who were from the same city and communicated in the native language of Portuguese. Questions focused on experiences with waste picking, safety, access to treatment, and ideas for improvement in the sector. Multiple participant perspectives were sought and females and males of various ages were all included in the interview process. Peer debriefing and review occurred before and after the development of interview questions and analysis of answers. Reflexive journaling and field notes occurred in the field diary, which was used to report on questions related to field immersion and impressions of each visited location. Supra-segmental features such as pauses and silences were identified with a “+” in parentheses, vowel elongation was represented by colon; these behavioral aspects can improve the analysis of critical discourse. Nonverbal resources, such as laughter, were also provided in the transcripts and were represented by double parentheses that were used to insert a comment on something that occurred during speech.

All interviews were recorded, transcribed, and later analyzed. Codes were manually generated based on the conceptual framework of a phenomenological study as described by Moustakas and from the research questions.^27^ Codes allowed the researcher to provide direct and indirect information, such as descriptions and quotes, to support themes and patterns. Codes were generated and made into a codebook; some examples of codes included “safety,” “medical waste,” and “treatment.” These characteristics were reviewed for themes and patterns to help understand the experiences of the subject, as well as perceptions and other phenomena. Themes were then generated from the codebook; these themes represented information supporting the experiences of waste pickers sorting through medical waste in open air dumps. Themes were then re-analyzed and verified by the research team.

## Results

A total of 28 females and 6 males (n=34) were interviewed by the Brazilian research team (e.g. principal investigator, additional researchers, and graduate school students). The participants were garbage collectors who actively worked at the dumpsite. A convenience sample was taken, although more females were interviewed because the sector is comprised of a greater number of women waste pickers. Most waste pickers were between the ages of 30 to 39 years old (41%). Total time spent working as a waste picker ranged from 7 months to 40 years; work hours averaged 8 hours per day at 6 days per week. In general, most participants had low education levels (32% were illiterate) and did not obtain a formal education (49% received an elementary education and 17.5% went to high school). Most waste pickers racially identified as “parda” (63%), which is mixed Black and White, with 25% identifying as Black and 11% identifying as White.

Through these interviews, substantial information was gathered to provide an accurate description of recyclable collectors' exposures to hazardous medical waste. Furthermore, participants' answers were similar for each question and did not introduce any additional information, confirming saturation of the study questions.^27^ The participants' answers highlighted environmental exposures that collectors were often faced with while working at the waste site. Key concepts that developed and arose during the research analysis became the main themes; these themes focused mainly on continued exposures and hazards in an industry that has effectively become illegal in Brazil *(Supplemental Material)*. This information suggests that both major and minor hazards are likely to continue despite waste site closures.

### Physical and biological exposures

Occupational accidents experienced by recyclable collectors typically occur because of the hazardous nature of the work environment. Interviews detailed many accidents with sharp objects. These accidents occur because medical waste often contains syringes, scalpels, and needles. Syringes appeared to be the most common physical exposure for recyclable collectors.

*“I've been punctured here several times—not just once. Here, everyone gets punctured…”* (Participant E-28)

Some participants spoke about the presence of syringes with the needle attached without the protective cover, not only in medical waste, but in general trash as well. In general, this was found to be common practice in hospitals, as employees and patients alike mistakenly mix biological, chemical, and common waste together.^28^–^32^ These items caused many injuries to the workers, especially to the hands and upper extremities, as well as near the feet and legs. Workers explained that “there are a lot here; I've already [cut] myself with a syringe” and that they had “already cut [their] finger”, suggesting that these injuries were commonplace and had occurred on the day of the interview.

*“… I punctured myself with a syringe because it came in an ordinary [white trash bag from the supermarket].”* (Participant E-9)

According to standards set forth by the Brazilian legislation, common waste should be appropriately packed in black bags or according to regulations set forth by each district. Sharp objects should be packed in hard-walled boxes with the infectious symbol visually placed on the outside; these boxes must be leak-, puncture- and burst-resistant. While most recyclable collectors are aware of these safety procedures, some participant responses confirmed that they were unaware of the proper packaging and disposal of this type of waste. Other studies confirmed that syringes are often placed in hard plastic bottles or milk cartons.^28^,^30^,^31^ These discrepancies can make it difficult to avoid hazards because of inconsistencies in packaging and waste bag colors.

*“They come in bags of all colors (+)… we know that when it's white, it's from the hospital. But the problem is that [syringes] come in bags of other colors too. Here, they also come in plastic bottles with the syringes inside; they also come in milk cartons.”* (Participant E-31)

Recyclable collectors were also exposed to biological waste such as body parts, urine, blood and serum. “*I've found medications and even pieces of people, in fact this is common here.*” (Participant E-1) Other participants confirmed these biological exposures and provide more detail on the types of waste. “*… I have also seen colleagues get contaminated with blood bags and tubes of serum.*” (Participant E-12). Other participants provided information on occupational biological waste exposures.

*“We find all the [stuff] that [you] cannot even imagine. [We find] junk like small fetuses and dead people. And [other times], [we find] dirty things with blood, urine sample containers, umbilical cords, and placentas full of blood.”* (Participant E-24)

### Slips, trips, and fall hazards

With workers focused on sorting, this could present a hazardous situation that could result in injury or fatality due to falls, trampling, and burial by waste. Especially in landfills, this situation can arise when recyclable collectors compete for space with tractors and trucks while removing and sorting garbage at the waste site.

*“... we are in a hurry—there is no time to talk, because if we talk, inattentively, the machine [can run us over] (+). There was a friend of mine who's dead because the guy at the machine did not see her.”* (Participant E-16)

This situation was also confirmed by another participant who explained that she was also hit by a tractor when she was not paying full attention to it.

*“… I was hit by a tractor [and it] crushed my leg… I was not paying attention [and then] when I [finally] saw it, [my leg] was crushed. [Afterwards], I fell into the slurry pond… I just did not die because my daughter—[when] I sank down [in the water up] to my neck—she saw me and pulled me out.”* (Participant E-23)

In fact, from 2009 to 2014, there were 14 accidents involving recyclable collectors in “Lixão da Estrutural” and 11 were related to trucks and tractors.^33^

### Personal protective equipment

One way to reduce exposure of individuals to risk agents is to use PPE. This equipment could include gloves, goggles, sun-protection, hardhats, protective clothing, and more. It was expected that some type of protection would be used by recyclable collectors in order to reduce harmful exposures. In addition, it is also the responsibility of the Brazilian Ministry of Labor to establish rules and regulations regarding workforce health, while considering the unique features or tasks of each sector (see: Article 200 of the Consolidation of Labor Laws). Under Brazilian regulations, individuals are required to wear equipment that protects the head, eyes and face, upper limbs, lower limbs, core, skin, ears, lungs, and that provides protection from falls.

In the present study, PPE was reportedly used by most of the participants. However, the equipment was inadequate and did not protect them from the danger of contact with sharp objects. Participants reported using rubber gloves which are not suitable for the risks to which the recyclable collectors were exposed because “*they tear easily*” (Participant E-30). The specific type of gloves was not described, so that information is not provided.

*“... this one time, I felt that there was something strange… when I pulled out my hand, there was a syringe stuck in my glove. So then, I took off the glove [to remove it] and blood was coming out of my finger. I was so afraid, I started to tremble (+), but I kept working.”* (Participant E-24)

Cost and limited provisions were barriers to obtaining and using appropriate equipment for protection.

*“… here we receive two gloves every 15 days; if one rips, we use the other.”* (Participant E-30)

*“The gloves are expensive, the glasses are expensive. When they do provide us [with gloves or glasses], [they only give us] a few [pairs] or [sometimes] do not give them to us [at all].”* (Participant E-11)

### Healthcare and homemade remedies

Some collectors understood the biological hazards associated with sharps punctures and actively tried to minimize the risks associated with sharp objects. Unfortunately, sometimes these solutions did not work, and waste pickers were wounded as a result. Subsequently, individuals often resorted to self-treatment for injuries. Most of these solutions were homemade remedies, such as saltwater and other concoctions.

*“When I opened [and reached in the trash bag], I punctured my finger... So, I pressed the finger [to stop the blood] and put [a mixture of] cachaça, lemon and salt on it—some people do that here.”* (Participant E-11)

These individual solutions result in individuals not accessing adequate preventive measures, such as the use of vaccines, serums or prophylactic drugs. Some participants reported that seeking medical care would result in “lost time”, as the time spent waiting for care in a healthcare setting would mean lost work time at the waste site, resulting in less pay. Others reported that there were significant delays in care as well as social stigmatization on behalf of the health professionals who treated them.

*“... yesterday I got punctured with a needle. It didn't have blood [on it]... I think it was serum. But I did not seek assistance. … We do not even have time to go, because when we get [to the hospital] it takes too long to be seen [by a physician].”* (Participant E-10)

Instead of receiving care at a hospital, collectors self-medicated or received medicine through a pharmacist.

*“I poked the tip of my finger with a hospital needle... look at my swollen finger. (+) [My friend] told me to go to a hospital and I said, I'll go to the pharmacy [instead] and buy medicine there [because] the pharmacist will give me the medicine.”* (Participant E-24)

Another solution to avoiding needle pricks was to throw the needles “into the bushes” and at least avoid personally stepping on them.

*“Sometimes the needles are facing upwards and it is the worse, because it sticks in the foot of the colleagues.”* (Participant E-11)

### Use of collected medical waste

Some collectors gathered or saw other workers gathering medicine from waste for personal use or to sell. These medications included diabetes medication, inflammation or pain pills, vaginal ointment, and cancer medication. One reason mentioned for using these drugs was “*out of necessity*” (Participant E-34). The availability of the medication and the timeliness of encountering it appeared to be major reasons for the decision to selfmedicate. “*I used a vaginal ointment that was sealed. I cut myself and used it [as an ointment] on the cut.*” (Participant E-2). Other participants reported taking advantage of the convenience of found medication, especially to aid with health issues they experienced while working at the dumpsite. “*When it's not out of date, yes [I take it]. What I found was an anti-inflammatory for pain… I have a lot of pain here in [my] back.*” (Participant E-28)

Other participants reported profiting from finding medicine and then selling it. “*Some people take the expired drugs or also sell [them]; sometimes, they [even] find sealed packages to sell.*” (Participant E-8)

### Perception of risk

Many workers understood the danger posed by encountering medical waste, particularly because of their potential exposure to contaminated syringes. However, they lack knowledge of all of the risks and consequences associated with exposure to medical hazards.

*“…we do not have much knowledge, but we are thinking about the patients who use it [the syringes], if it comes contaminated and we are stuck, it is a huge risk. …the work we do is already very harmful.”* (Participant E-22)

Another participant described how exposures might affect the health of workers in this sector. “*I think it's bad [being exposed to medical waste] and sometimes it's dangerous, but sometimes it's not dangerous.*” (Participant E-20). This perception seemed to indicate a neutral attitude towards waste exposures and their effects, despite knowledge of some of the hazards involved.

*“[I know people] who use syringes that have AIDS. We [should] get scared, right? [But, we don't know if syringes] are contaminated or not.”* (Participant E-21)

## Discussion

Recyclable collectors are a group of individuals who make a living by sorting through waste to find recyclable goods, which are then traded in for money. The waste presents many occupational hazards (e.g. needle sticks), and exposures occur when sifting through garbage. Previous research has reviewed health outcomes among collectors, but has primarily focused on solutions to prevent these hazards, such as PPE, instead of determining occupational exposures.^34^,^35^ The present qualitative research study sought to understand the hazards, medical exposures, risks, and treatment choices directly from waste pickers.

**Figure 1:**
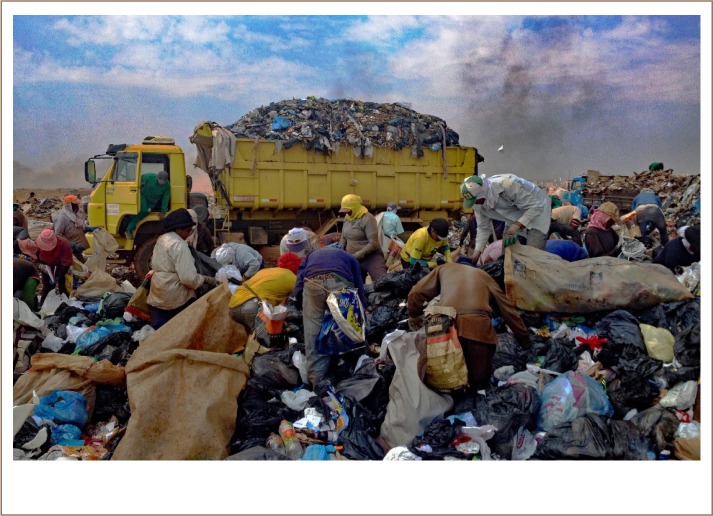
Recyclable waste collectors sorting through waste in Estrutural, Brasilia, Brazil, June, 2016. Published with permission.

In addition, since this site was officially closed in late 2017, it is important to highlight the working conditions of a population that is not currently supported by national workforce governmental standards or treated as a recognized working class. Participant responses provide further insight on specific occupational exposures that continue to be threats for recyclable collectors, ways in which workers address these hazards, barriers to accessing healthcare or treatment, and suggestions for improving work conditions *(Figure 1).* Since this type of work continues despite governmental shutdowns of sites, it is imperative that other organizations (e.g. academic, nonprofit, etc.) work to create safer work environments for waste collectors. Recyclable collectors are a population that is particularly vulnerable to occupational and environmental hazards, and are therefore susceptible to accidents and the effects of work site exposures.^36^

This research identified key information on the type of hazards that collectors may be exposed to, especially regarding medical waste and wounds from syringes. Hazards primarily included pricks from needles and syringes. These pricks can cause exposure to a variety of biological hazards (e.g. HIV/AIDS).^37^–^39^ There are multiple reasons for these exposures, including worker complacency, as well as inadequate disposal of medical waste in common garbage, that put collectors at risk. This information confirms the need for solutions to mitigate these types of exposure.

One important mitigation step is the use of PPE. However, the use of such equipment does not completely prevent the occurrence of accidents, it only minimizes direct contact of collectors with hazardous waste. In an earlier study conducted in the Federal District, it was found that the frequency of occupational accidents was higher among those who reported using some type of PPE, and one reasonable explanation could be due to the quality of the equipment (e.g. old disposable gloves) used by collectors.^40^ Most recyclable collectors reported using some type of PPE, but others gave reasons for not using this equipment, such as the fragility of gloves against contact with sharp objects, difficulty of handling waste materials, the high cost of appropriate gloves, and limited provision of equipment by the government. Requiring recyclable collectors to wear PPE (e.g. sturdy gloves, hats, boots, long-sleeved clothing, goggles) does not solve the problem of hazardous working conditions at dump sites, however, providing more education and monitoring may contribute to increased worker safety. The government of Brazil supports the use of gloves by distributing them to waste pickers, but this outreach could by improved by adding more equipment and education to encourage the use of PPE.

Second, this informal occupation should be treated as an actual profession. This has been partly implemented by the government, as official sorting facilities, known as selective collection centers, have uprooted many waste pickers from informal open-air dumps to formal work settings. These actions have occurred because of Brazil's adherence to the Business Commitment to Recycling regulations (Compromisso Empresarial para a Reciclamen); as a result, fences have been built around open dump sites, entrances have been identified, slurry and burn off gas has been drained, and landfill sites have been established.^41^ This type of movement may help to ensure equality (e.g. access to healthcare and standards) set forth by governmental regulations. By acknowledging recyclable collection as a profession by the government, one major gain is that workers could have improved access to and better treatment from healthcare services, which was reported as an issue by the recyclable collectors.^42^ As reported by respondents, workers who had accidents with sharp objects often did not seek medical care or testing, possibly demonstrating a lack of education about the risks of contracting diseases or the denial of risks. Respondents also mentioned that they often do not seek care because they do not want to take time off from working, as this would lead to lost wages.^42^ This can be addressed by creating an official space for waste pickers to work.

Third, initiatives need to be developed to encourage people to practice proper disposal of health services waste. Currently, only 10.5% of medical waste with sharps disposal are stored appropriately in plastic containers.^28^ Correct disposal practices should be taught and encouraged by health services professionals, as they seek to increase safety in the handling of these types of waste. In particular, healthcare waste should be segregated and disposed of separately from common waste. There are programs in place throughout Brazil that encourage safe disposal practices. In Jaraguá do Sul, Santa Catarina, the Basic Health Units promote campaigns to educate insulin users about the importance of proper disposal of these materials.^43^ In Uberaba, Minas Gerais, patients are advised to deliver needles and syringes in appropriate industrialized containers or use packaging that has a lid, such as a powdered milk can, a mayonnaise container, or plastic bottles.^44^ The “Conscious Disposal” program has expanded its waste collection services; in Porto Alegre, new points of collection of sharp objects have already been installed. The program provides insulin users with a home collector that must be stored in a safe place and then discarded in specific collectors provided by the program.^45^ However, there are barriers that prevent significant advances in medical waste disposal, including lack of governmental pressure to administer these changes, lack of attention by public authorities on health issues in the waste sector, lack of technical training on solid waste management and urban cleaning systems, and few research centers examining issues of municipal solid waste.^37^ The current programs working to make medical waste disposal safe could be reviewed, enhanced, and redistributed to decrease hazards in target groups, such as waste pickers.

Possible limitations to the study included social desirability and translation errors. Social desirability is a bias that suggests participants may respond in a manner that is suitable to the researchers, seeking to be viewed favorably. This may result in under reporting to avoid persecution or pity from the researcher. Translation errors could also have occurred when the transcript was translated from Portuguese to English. In addition, the interviews were conducted in one specific location, and therefore, information that was gathered may not be generalizable or transferable to all people who work in dump site waste collection. Finally, non-response bias should be noted, as people who participate in research are inherently different from those who do not, and this may have affected the results.

As the study site is the largest waste site in Brazil, the present study provides insight into similar or smaller scale dump sites around the world. The results may be generalizable to workers who experience similar types of waste collection hazards, such as E-waste sifters who may encounter sharp wires.

Future research could examine differences in recyclable collectors versus special materials collectors as well as gather quantitative data in order to quantify the results of the present study. This information could provide a more comprehensive view on hazards experienced by waste pickers around the world.

## Conclusions

Informal waste management in Brazil has officially come to an end with the formal closing of all dumpsites throughout the country. The Brazilian government has created formal working spaces for garbage sorting and distributes PPE (e.g. gloves). Unfortunately, despite these actions, recyclable waste collectors continue to work and face occupational hazards associated with their informal occupations. One major hazard encountered by recyclable collectors is encountering sharp objects while sorting. These sharp objects, typically from medical waste, can result in physical injury and biological exposures. Waste collectors are at risk due to improper waste disposal at healthcare facilities, a need to work quickly to avoid other more serious hazards (e.g. being run-over by dump trucks), complacency or poor education on the use of proper PPE, and lack of acknowledgement of the profession by government officials and healthcare workers (e.g. physicians). The results of the present study confirm the barriers surrounding recyclable collectors to seeking treatment for injuries associated with garbage sifting. Recyclable collectors often face social stigmatization and are frequently overlooked by policy measures to improve the working conditions and livelihood of this population.

Measures to mitigate these occupational exposures include providing proper PPE, improving access to healthcare, increased education on workplace hazards (e.g. biological risks), and government support for upholding occupational health and safety standards in this work setting. Unfortunately, it is unlikely that there will be increased government support in this setting, as many of these sites are now illegal or officially “closed” in Brazil. As is apparent by the continued work of recyclable collectors, these closures do not impede workers from continuing using these sites, as it remains one of the only livelihood options for many people.^1^ To change this scenario, workers must become organized and demand governmental support and social protection. However, because recyclable waste collectors have not historically demanded these rights, the government should be aware of the benefits than can occur from formalization or legalization of recycling activities. Legalizing and regulating recyclable waste collection can aid in reducing poverty through maintained jobs, decreased costs of recycling for municipalities, and conserved and protected natural resources.^1^ Other areas that can aid in creating changes in this space include organizational support. For example, Women in Informal Employment: Globalizing and Organizing is a network comprised of individuals and institutions that use data and research to provide services or shape policies to help empower workers in informal industries. This organization has worked with waste pickers in Brazil to create frameworks for hiring waste pickers outside of informal employment, negotiated with policymakers and municipalities on improving waste pickers income and quality of life, and other areas of change.^13^ Addressing issues faced by waste pickers can improve many aspects of their lives. Ultimately, these findings demonstrate that it is of utmost importance to create a safe working environment for recyclable collectors worldwide to address these occupational hazards and to foster fair labor standards.
