# Supplementary material for: Continued Medical Waste Exposure of Recyclable Collectors Despite Dumpsite Closures in Brazil
Source: J Health Pollut. 2019 Jul 23;9(23):190905. doi: 10.5696/2156-9614-9.23.190905 (PMC6711331; doi:10.5696/2156-9614-9.23.190905)
Supplement: Supplementary file 1 [file Zolnikov_Supplemental.docx]

**Supplemental Material

Overview of work conditions and reasons for accidents or conditions**

| **Variables related to work conditions and accidents** | **Known reasons and findings for work accidents** | **Participant responses on work conditions and accidents** |
| --- | --- | --- |
| **Physical and biological**  **exposures** | -Work accidents experienced by recyclable collectors typically happened because of the precariousness of the work environment and the lack of suitable conditions.  -Interviews provided evidence of many accidents with sharp objects; medical waste is composed of syringes, scalpels, and needles.  -Syringes appeared to be the most common physical exposure that injured recyclable collectors. | General punctures; syringe punctures; hidden syringes and other items; exposure to feces, urine, and blood. |
| **Focused on more extreme hazards** | -In this environment, if collectors paid too much attention to avoiding exposure to medical waste, they might miss other hazards.  -Other hazards include falls, trampling, and even being buried by waste. | No time to worry about safety; fast life; tractor hazards. |
| **Personal protective equipment** | -Personal protective equipment is one way to reduce the exposure of individuals to risk agents; this equipment could include gloves, googles, sun-protection, hardhats, protective clothing, and more.  -It was expected that some type of protection would be used by the recyclable collectors in order to reduce harmful exposures. | Rubber gloves tear; syringes poke through gloves; only use one glove; expensive; incorrectly used; poor access. |
| **Healthcare and homemade remedies** | -Some collectors realized the biological hazards associated with sharps punctures they were exposed to and actively tried to minimize the risks associated with sharp objects; unfortunately, these solutions were often inadequate and there was little evidence of effectiveness in preventing harm. | Homemade remedies; serum; no time for care; need to work and make money; rely on pharmacists and not physicians; improper medical waste disposal. |
| **Use of collected medical waste** | -Some collectors gathered or saw other workers gathering medicine from waste for personal use or to sell.  -These medications included diabetes medication, inflammation or pain pills, vaginal ointment, and cancer medication. | Used medicine in waste; expired medication thrown; used on wounds; pain killers; sell recovered medicine for money. |
| **Perception of risk** | -Many workers understood that the collection of medical waste is a dangerous job, particularly because of their potential exposure to contaminated syringes. | Risk is understood; money more important. |
